# Different photosynthetic responses to heat and light favour green and red over brown macroalgae in the mediterranean sea

**DOI:** 10.1038/s41598-025-28235-8

**Published:** 2025-11-26

**Authors:** Leonie Hesse, Merlin Weiss, Selma D. Mezger, Yusuf C. El-Khaled, Benjamin Mueller, Alexandra Kler Lago, Mischa Schwarzmeier, Christian Wild

**Affiliations:** 1 Department of Marine Ecology, Faculty of Biology and Chemistry, University of Bremen, Leobener Str. 6, 28359 Bremen, Germany; 2 Institute for Biogeochemistry and Pollutant Dynamics, Department of Environmental Systems Science, ETH Zürich, Universitätstrasse 16, Zurich, 8092 Switzerland; 3 Division of Biological and Environmental Science and Engineering (BESE), King Abdullah University of Science and Technology (KAUST), Thuwal, Saudi Arabia; 4 Institut für Marine Biologie (IfMB), Karlsruhe, Germany

**Keywords:** Macroalgal physiology, Stress responses, Oxygen flux, Climate change, Community shifts, Ecological plasticity, Ecology, Ecology, Ocean sciences, Plant sciences

## Abstract

Global change is rapidly altering temperature and light regimes in marine environments, yet the physiological responses of benthic primary producers to these shifts remain poorly understood. Red (*Phyllophora*), brown (*Cystoseira*) and green (*Flabellia*) macroalgae dominate distinct assemblages in the Mediterranean Sea and act as ecosystem engineers that overgrow rocky substrates or seagrass meadows. Their taxon-specific physiological responses to changing temperature and light levels may have long-lasting consequences on algal community composition and overall ecosystem functioning, but remain poorly understood. Here, we experimentally test how water temperature and light availability affect photosynthesis and respiration in these macroalgae using specimens collected on Giglio Island, Italy. We exposed different macroalgae to a full-factorial combination of three temperatures (21, 26, and 30 °C) and light intensity levels (180, 320, and 760 µM photons m^− 2^ s^− 1^) and measured oxygen fluxes to determine net photosynthesis (P_n_) and respiration (R). Temperature and light both significantly influenced net photosynthesis, with marked taxon-specific responses. In *Cystoseira*, photosynthesis nearly ceased in high light and high temperature conditions, also indicated by a critically low photosynthesis to respiration (P:R) ratio. *Flabellia* and *Phyllophora* maintained more stable photosynthetic rates under medium light and temperature conditions and showed a moderate decline in their P: R ratios. *Flabellia* sustained slightly higher photosynthesis rates in high light and temperature conditions than *Phyllophora* and both significantly outperformed *Cystoseira* under maximal light and temperature conditions. Our findings suggest that under increasing temperature and light regimes, *Cystoseira* may decline, *Phyllophora* may persist, and *Flabellia* may even thrive. This may in turn degrade habitat complexity and affect coastal community assembly, as *Flabellia*, unlike the architecturally complex and persistent *Cystoseira*, does not form stable, three-dimensional habitats for associated organisms.

**Supplementary Information:**

The online version contains supplementary material available at 10.1038/s41598-025-28235-8.

## Introduction

Climate change is increasing water temperatures and light availability in many marine systems, particularly in semi-enclosed basins like the Mediterranean Sea^1^. The frequency and intensity of marine heatwaves in the Mediterranean Sea have risen sharply^2^, causing mass mortalities in over 50 macrobenthic taxa across eight phyla from surface waters down to 45 m depth, with Cnidaria, Bryozoa, and Rhodophyta most severely affected. These events caused major structural and functional disruptions in benthic communities, especially affecting unique keystone species that build complex three-dimensional habitats^3^. Concurrently, the thermocline (i.e., the transitional layer between water masses of differing temperatures), typically positioned between 10 and 30 m depth, is expected to form earlier and persist longer under future warming scenarios^4^. This may lead to increased water column stratification and limit vertical nutrient flux, resulting in nutrient depletion in the surface layer^5^. Consequently, phytoplankton growth may be supressed, leading to increased light penetration and overall higher light levels at greater depths^6^. Local anthropogenic stressors such as eutrophication, which leads to filamentous algal blooms, as well as coastal development and land-use changes, which increase sediment runoff, may further reduce light availability^7,8^. Therefore, both temperature and light regimes are expected to undergo substantial shifts in the near future due to a combination of climate change and local disturbance effects^9,10^.

Temperature and light are also among the most critical environmental drivers that control physiological processes in phototrophs. For example, net photosynthesis typically increases with temperature up to a taxon-specific optimum beyond which enzyme denaturation and rising respiration can result in net carbon loss^11,12^. This balance is often expressed as the photosynthesis-to-respiration (P: R) ratio, which serves as a proxy for metabolic performance^13^. Under low light or high temperature, P:R ratios may fall below 1, indicating reduced photosynthetic capacity and potential carbon deficits^14^. Excess light stress can further reduce photosynthetic capacity via photoinhibition^15^. However, some macroalgae, especially opportunistic macroalgae species like *Ulva lactuca* and *Cladophora* spp., can benefit from elevated light and temperature by enhancing carbon assimilation and growth, demonstrating a high degree of physiological plasticity^16^. Both temperature and light thereby define the abiotic niche spaces of macroalgae and their capacity to form and sustain habitats^17^. Taxon-specific differences in metabolic responses to changing light and temperature levels are therefore likely to cause community shifts in benthic primary producers, such as macroalgae^18^.

Macroalgae are among the most abundant benthic primary producers in coastal systems, modifying habitat structure and supporting diverse assemblages of invertebrates, fish, and epiphytes^10^. In the Mediterranean Sea, functionally distinct taxa occupy different ecological niches. For example, the red algae *Phyllophora* form dense mats on rocky substrates, creating complex microhabitats that attenuate currents and light penetration^19^, support a high diversity of invertebrates^20^, and enhance overall benthic biodiversity^21^. The brown alga *Cystoseira* forms underwater forests, with canopies typically reaching 20–30 cm in height, whose three-dimensional structure enhances biodiversity by providing essential habitat for invertebrates, fish, and epiphytes^22^. The green alga *Flabellia* inhabits well-lit hard substrates and typically occurs within phytocoenoses, i.e., structured plant communities comprising the collective vegetation in a particular habitat, often alongside *Posidonia oceanica* meadows^23^. Unlike low-lying mat-forming algae or upright forest-forming taxa, *Flabellia* forms a more dispersed, patchy cover close to the substrate surface^24^. Despite its limited physical structure, it enhances benthic stability and hosts diverse epibiontic taxa, including calcareous algae and tube worms^25^. However, compositional shifts in these predominant macroalgal assemblages are becoming increasingly evident: Large, canopy-forming macroalgae are replaced by opportunistic or turf-forming species and/or invasive taxa such as *Ulva* spp. or *Sargassum muticum*, which displace native communities by forming dense overgrowths^26,27^. Also, *Phyllophora* and *Cystoseira* were observed to overgrow seagrass meadows and hard-bottom habitats, causing a transition toward simplified, algal-dominated assemblages^28^. These observed compositional shifts have been proposed to be associated with changes in environmental parameters including temperature, light availability, and eutrophication, yet underlying taxon-specific differences in the physiological response of macroalgae assemblages to these parameters are largely unknown.

Here, we investigated the physiological performance of three macroalgae assemblage types, the red algae *Phyllophora*, the brown algae *Cystoseira*, and the green alga *Flabellia*, to a combination of different light and temperature levels. We quantified net (P) and gross photosynthesis, respiration (R), and the P: R ratio at a combination of three different light and temperature levels following a full-factorial design.

Specifically, we asked (i) how temperature and (ii) light availability influence oxygen fluxes; (iii) and whether these effects interactively affect the oxygen fluxes in *Phyllophora*, *Cystoseira* and *Flabellia*. We hypothesised that rising temperatures would reduce net photosynthesis and photosynthesis-to-respiration (P: R) ratios, with taxon-specific variation reflecting differences in thermal optima and metabolic flexibility. Similarly, we expected photosynthetic performance to increase with light availability up to a taxon-specific threshold, beyond which photoinhibition would impair carbon assimilation. Finally, we expected temperature and light to interact, with combined stress amplifying metabolic impairment and favouring stress-tolerant taxa; yet the observed responses to differ among species, reflecting distinct physiological sensitivities.

## Material & methods

### Study site

We conducted the experiment in September 2021 at the laboratory facilities of the Institute for Marine Biology (IfMB) on Giglio Island, Italy, and sampled macroalgae assemblage by scuba diving at the Punta del Fenaio dive site (42°23’19.98”N, 10°52’47.92”E) on the island’s northwestern coast, within the Tuscan Archipelago National Park, Tyrrhenian Sea. Sampling occurred at depths of 20–22 m, where *Phyllophora* and *Cystoseira* co-dominate the benthic community, and *Flabellia* grow on nearby hard substrates. All specimens were collected within a 10 m radius to ensure that individuals were exposed to similar environmental conditions, allowing for interspecific comparisons under controlled laboratory conditions.

### Data collection and experimental design

**Fig. 1:**
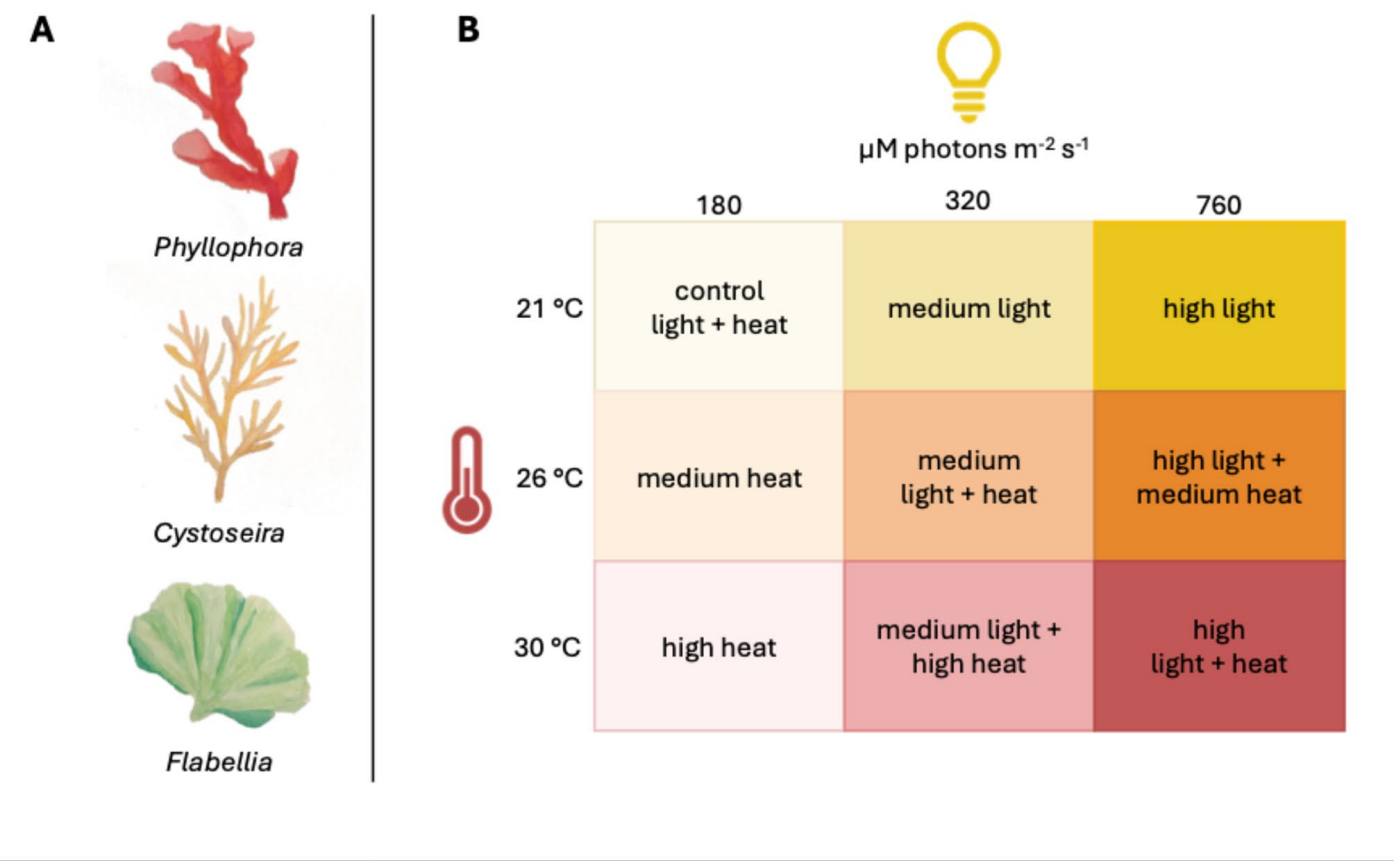
Experimental design of the factorial incubation experiment. (**A**) The three Mediterranean macroalgal taxa studied: *Phyllophora*, *Cystoseira*, and *Flabellia*. (**B**) Crossed experimental treatments combining three light intensities (180, 320, 760 µmol photons m^−2^ s^−1^) and three temperature levels (21 °C, 26 °C, 30 °C). Each square in the matrix represents one of the nine light × temperature treatment combinations applied during oxygen flux incubations. Labels denote simplified condition names used in subsequent figures.

We opportunistically sampled of *Phyllophora*, *Cystoseira*, and *Flabellia* specimens from macroalgae assemblages on rocky substrates. While morphological traits indicated that individuals discussed as *Phyllophora* and *Flabellia* represented *Phyllophora crispa* and *Flabellia petiolata*, species-level identification was less certain for *Cystoseira*, where more than one species may have been present. We assessed whether pooling at the genus level masked potential within-taxon heterogeneity by comparing dispersion metrics and found the variability in *Cystoseira* to be comparable or lower than that of the other taxonomically resolved algae. To ensure consistency and to focus our analysis on functional differences among taxa with respect to pigmentation, we therefore retained all samples at the genus-level for subsequent analyses and accounted for potential within-genus variability. Each sample was carefully detached and immediately transferred into 2 L Kautex jars filled with ambient seawater. Upon return to the laboratory, all samples were placed into flow-through husbandry tanks and maintained under stable conditions (21 °C, 12:12 h light/dark cycle) with light levels comparable to measured in situ conditions. We conducted incubation experiments to quantify metabolic rates (net photosynthesis and respiration) under control conditions (21 °C, 180 µmol photons m^−2^ s^−1^) and under two elevated temperature treatments (26 °C and 30 °C) and two elevated light treatments (320 and 760 µmol photons m^−2^ s^−1^) (see Fig. 1).

### Metabolism measurements

All metabolism incubations were conducted ex situ and within 3 days after sample collection. For the O_2_ flux measurements, incubation chambers (560 mL volume) were filled exclusively with ambient seawater collected the same day, and algal material was placed carefully inside the incubation chambers (*n* = 6 chambers for *Phyllophora*,* Cystoseira* and *Flabellia*, respectively). Additionally, four chambers without algal material served as seawater controls to correct for planktonic background metabolism. All chambers were sealed gas-tight and without any air enclosure. During the incubations, the chambers were randomly placed in four independent tempered water baths kept at treatment temperatures. Water temperatures were reached prior to the start of the experiment at respective light levels before the chambers were closed. All chambers were constantly stirred at 200 rpm to ensure fully mixed conditions and avoid the build-up of concentration gradients. A 2 h light incubation was followed by a 1.5 h dark incubation. Incubation times were kept as short as possible to prevent excessive CO_2_ consumption or production. Dissolved oxygen levels were measured immediately before and after each incubation using a WTW Multi 3430 multimeter equipped with a WTW DFO 925 dissolved O_2_ sensor. Oxygen levels remained above 50% saturation in the dark and below 120% in the light, thereby avoiding pronounced hypoxic or hyperoxic conditions that could have affected algal physiology.

Measured dissolved oxygen (O_2_) concentrations from light and dark incubations were used to calculate net primary production (P_net_) and dark respiration (R_dark_), respectively. O_2_ start concentrations were subtracted from end concentrations and normalized to incubation time. O_2_ fluxes were corrected for the seawater control signal, related to incubation volume, and normalized to the surface area of the incubated algal sample. Surface areas of samples were calculated^29^. Each incubated algal sample was placed on a laminated grid paper and flattened with a glass pane without overlying algal thalli. Pictures were taken from the top at a 90° angle and the surface area of each sample was quantified through automated substrate selection using the Photopea online software. Values were then multiplied by two to consider both sides of the flattened algal sample. We then calculated the P: R ratio. Since the P: R ratio requires an estimate of gross photosynthesis (P_g_), we first derived P_g_ as the sum of net photosynthesis (P_n_) and the absolute value of respiration (R). The P: R ratio was then computed by dividing P_g_ by the absolute R value, as R was treated as a negative flux. To improve numerical readability, metabolic rates originally measured in µmol O_2_ cm^−2^ h^−1^ were converted to nmol O_2_ m^−2^ s^−1^ by multiplying by 2,777.78. This factor accounts for the change in amount (1 µmol = 1,000 nmol), area (1 cm^2^ = 1/10,000 m^2^), and time (1 h = 3,600 s), yielding a total factor of (1,000 × 10,000) ÷ 3,600.

### Statistical analysis

We analyzed a total of 54 *Cystoseira* (brown), 54 *Phyllophora* (red), and 53 *Flabellia* (green) macroalgae samples. Our setup included 6 samples per treatment and per taxa (with the exception of 5 *Flabellia* samples in control temperature and light conditions). To understand how photosynthetic performance changed across experimental conditions, we statistically modelled net photosynthesis and the P: R ratio in response to the different temperature and light levels. All analyses were done in R (version 4.5.0). Prior to analysis, we assessed data normality, linearity, homogeneity, and outliers^30^. As net photosynthesis and the P: R ratio both exhibited non-linearity with temperature and light, we treated these predictors as categorical factors with three levels: control (21 °C), medium (26 °C), and high (30 °C) for temperature, and control (180 µmol photons s^− 1^ m^− 1^), medium (320 µmol photons s^− 1^ m^− 1^), and high (760 µmol photons s^− 1^ m^− 1^) for light intensity.

To test the effects of temperature and light intensity on net photosynthesis across taxa, we first fitted a series of linear mixed-effects models using the R package lme4^31^. The initial model included a full-factorial structure with temperature, light intensity, genus, and all two-way and three-way interactions as fixed effects. The genus was initially included as a random effect to account for taxon-specific variation in responses to treatment combinations. Both random intercept and random slope structures were tested to account for baseline variability in net photosynthesis and differential taxon-specific responses. However, nested model comparisons using likelihood ratio tests implemented via ANOVA indicated that random slopes did not significantly improve model fit, and their inclusion risked overparameterization. Also, taxon-specific intercepts showed minimal variation (< 0.001), leading us to discard the random effect entirely and opt for a Linear Model to avoid convergence issues and numerical instability. This also suggested that genus-level classifications adequately captured functional differences within algal samples of each type, and that inferences drawn from comparisons between taxa are robust to potential species-level heterogeneity.

**Fig. 2:**
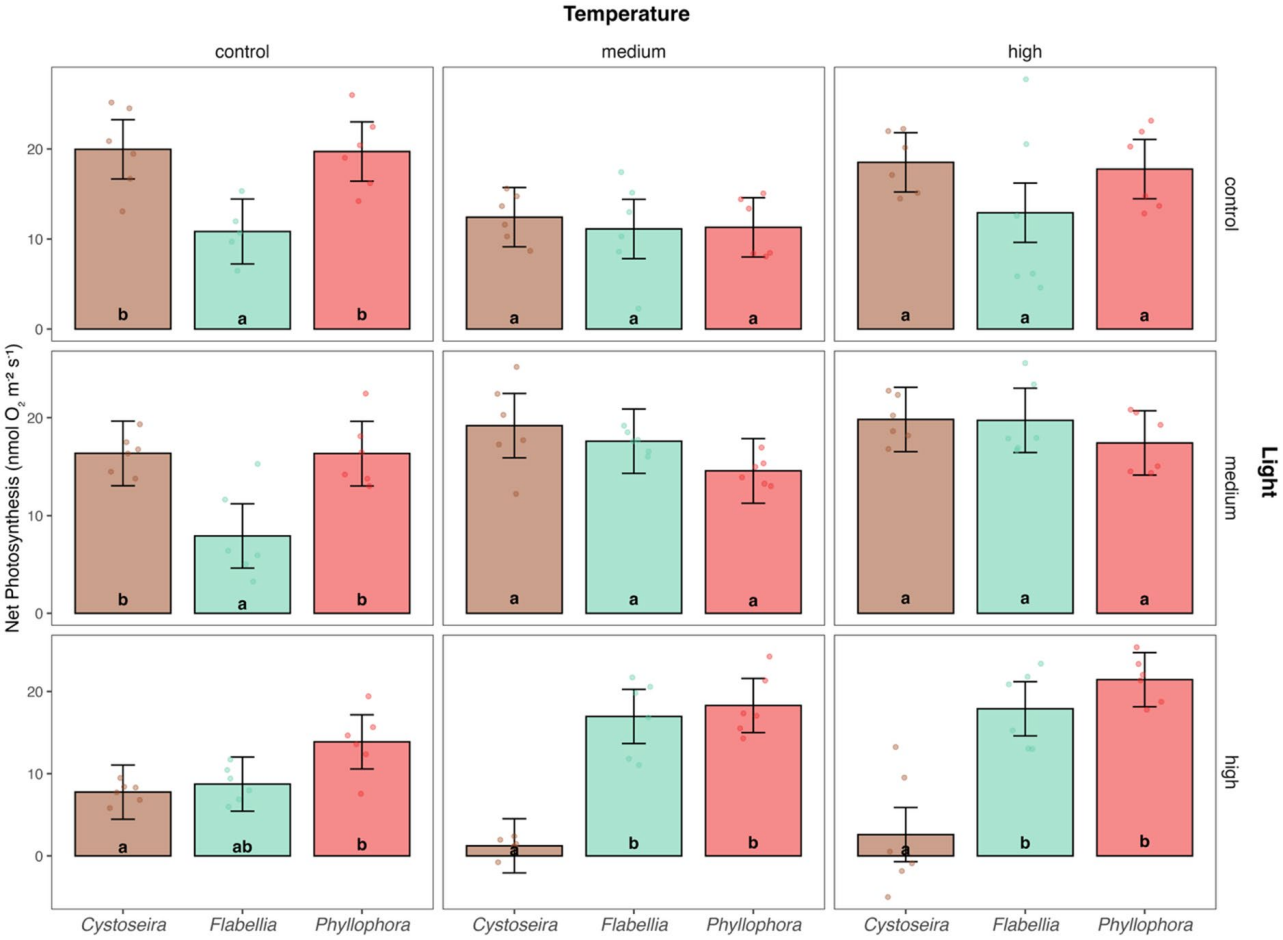
Estimated marginal means (EMMs) of net photosynthesis across all combinations of temperature (control, medium, high; horizontal grid) and light (control, medium, high; vertical grid) for three Mediterranean macroalgal taxa (*Cystoseira*, *Flabellia*, *Phyllophora*). Bars show model-estimated means from the full-factorial linear model with 95% confidence intervals (black whiskers), overlaid with raw data points. Letters within each panel denote results of post-hoc pairwise comparisons (Tukey adjusted) between taxa (significance level α = 0.05).

Model fit and performance were assessed using likelihood ratio tests and the Akaike Information Criterion (AIC). To ensure model parsimony, non-significant higher-order interactions were sequentially removed using backward stepwise selection, with model comparisons based on the AIC. However, reduced models performed significantly worse than the full-factorial model, and we therefore retained all fixed effects and interaction terms. Final model validation was done via residual diagnostics and by confirming no violations of homoscedasticity and multicollinearity. To assess the statistical significance of main effects and interaction terms, we conducted an analysis of variance (Type III ANOVA) on the final model, presenting F-values and associated p-values (Table 1). The final model showed a solid explanatory power (Adj. R^2^ = 0.603), accounting for 66.8% of the variance in net photosynthesis (see Supplementary Table S-1). Post-hoc pairwise comparisons of significant terms were done using emmeans^32^, with Tukey adjusted p-values to control for multiple comparisons (Fig. 2, see also Supplementary Table S-2). All tests were two-tailed with α = 0.05.

**Fig. 3:**
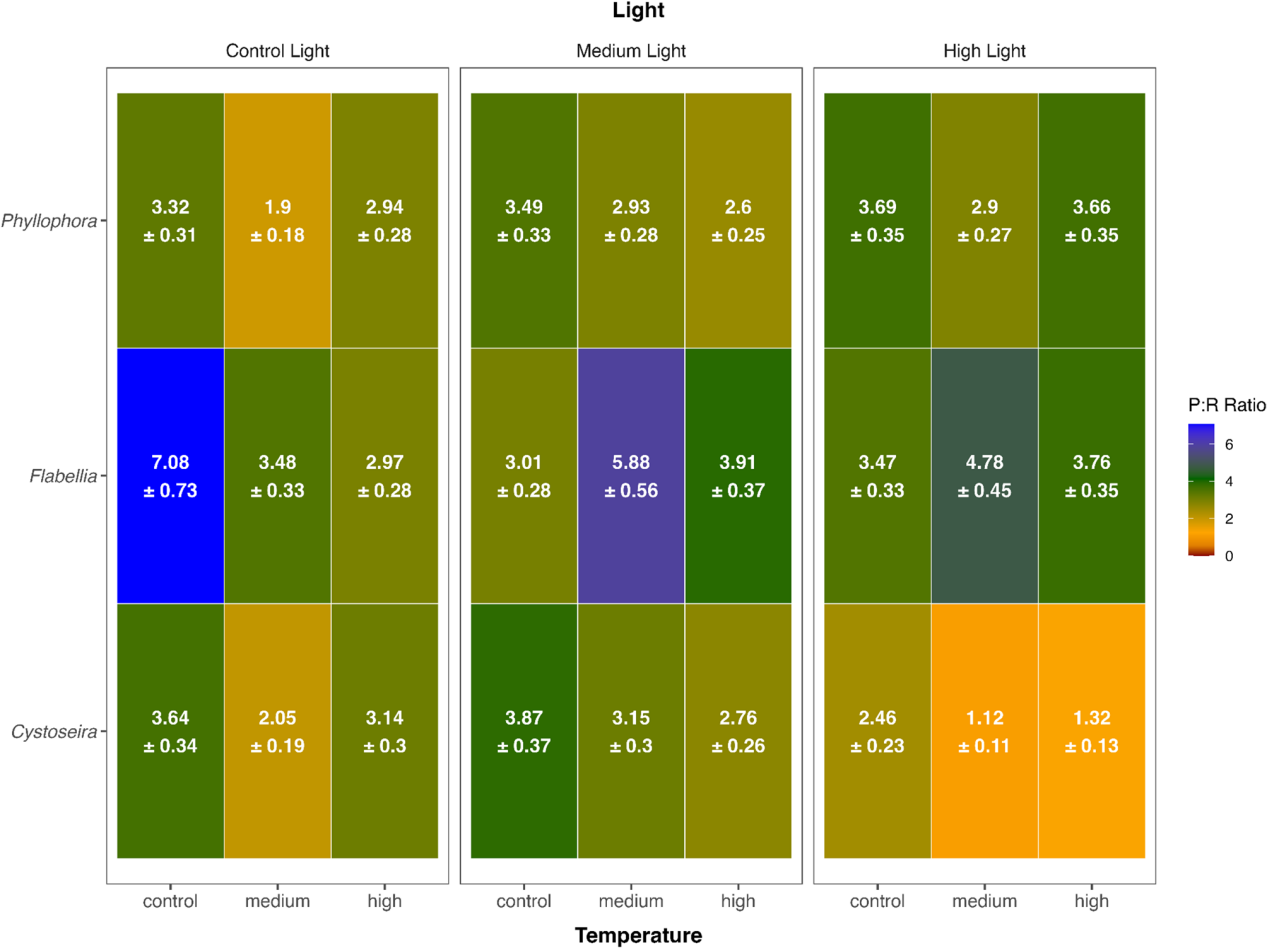
Heatmap showing the estimated marginal means (EMMs) of the P:R ratio for three algal genera across all combinations of temperature and light conditions. Colours indicate metabolic states: red (P:R < 1, metabolic imbalance), orange-yellow (1 ≤ P: R < 3, moderate autotrophy), green (3 ≤ P:R< 5, stable autotrophy), and blue (P:R > 5, strong autotrophy and high photosynthetic efficiency.

To assess the balance between photosynthetic oxygen production and respiratory oxygen consumption, and to identify conditions under which metabolic imbalance occurs, we modelled the P: R ratio following the same approach used for net photosynthesis. The most parsimonious model was a GLM with a Gamma distribution and log-link function, given the right-skewed distribution of P: R ratios and their strictly positive values. Temperature, light intensity, and taxon identity, along with their full-factorial interactions, were included as fixed effects. Model validation followed the same procedure as for net photosynthesis. While residual diagnostics indicated some deviations from the assumed Gamma distribution at higher P: R values, overall model estimates remained robust. Due to concerns regarding deviations from the assumed Gamma distribution at high values, we report estimated P: R ratios but refrain from formal inferences using p-values for statistical significance. Taxon- and treatment specific variation in P: R ratios was visualized descriptively using a heatmap (Fig. 3).

## Results

Photosynthetic performance in Mediterranean macroalgae responded strongly to combined warming and light stress, with taxon-specific patterns. A significant three-way interaction (Temp × Light × Taxon: F = 2.32, *p* = 0.023; Table 1) showed that net photosynthesis depended on the specific temperature–light combination within each taxon. Although temperature (F = 9, *p* < 0.001), light (F = 16.58, *p* < 0.001), and all two-way interactions (*p* < 0.001) showed significances, these effects are not considered separately, as the higher-order interaction governs the response.

**Table 1:** Type III analysis of variance (ANOVA) for the final linear model of net photosynthesis, based on 161 observations.

Effect	Df	F value	*p* value
Temperature	2	9	< 0.001
Light	2	16.59	< 0.001
Taxon	2	12.28	< 0.001
Temperature : Light	4	6.02	< 0.001
Temperature : Taxon	4	8.48	< 0.001
Light : Taxon	4	28.86	< 0.001
Temperature : Light : Taxon	8	2.32	0.023

Fixed effects include temperature, light intensity, and taxon identity, along with their two- and three-way interactions. All assumptions of normality and homoscedasticity were met. Interaction terms indicate deviations from additivity in treatment responses.

Overall patterns reflected this interaction but differed strongly among taxa. For *Cystoseira*, light was the dominant stressor: photosynthesis was already reduced under high light at control temperature (7.8 ± 1.7 nmol O_2_ m^−2^ s^−1^ vs. ~20 nmol O_2_ m^−2^ s^−1^ at control light Fig. 2; Supplementary Table S-1) and declined further with warming (1.2 ± 1.7 nmol O_2_ m^−2^ s^−1^ at 26 °C; 2.6 ± 1.7 nmol O_2_ m^−2^ s^−1^ at 30 °C; Fig. 2; Supplementary Table S-1). At control light, performance declined at 26 °C (12.4 ± 1.7 nmol O_2_ m^−2^ s^−1^) and returned to near-control levels at 30 °C (18.5 ± 1.7 nmol O_2_ m^−2^ s^−1^), a fluctuation within error and likely not indicative of recovery. *Cystoseira* showed the most severe impairment when high light and warming co-occured. In contrast, *Phyllophora* exhibited partial compensation: photosynthesis declined at 26 °C under control light (11.3 ± 1.7 nmol O_2_ m^−2^ s^−1^; Fig. 2; Supplementary Table S-1) and also rebounded to near-control values at 30 °C (17.8 ± 1.7 nmol O_2_ m^−2^ s^−1^; Fig. 2; Supplementary Table S-1), but then remained stable across temperatures at medium light (14.57 ± 1.67 at 26 °C vs. 17.42 ± 1.67 at 30 °C; Fig. 2) and increased under high light with warming, reaching 21.4 ± 1.7 nmol O_2_ m^−2^ s^−1^ at 30 °C (*p* = 0.006; Supplementary Table S-1). *Flabellia* showed the greatest resilience: although rates remained modest under control light (10.84 ± 1.83 to 12.92 ± 1.67; Supplementary Table S-1), warming substantially enhanced performance under medium and high light, increasing from 7.92 ± 1.67 at baseline to 17.59 ± 1.67 at 26 °C and 19.73 ± 1.67 at 30 °C (Fig. 2; Supplementary Table S-1). These taxon-specific trajectories underscore that responses cannot be attributed to temperature or light alone but emerge from their interaction: strongly negative in *Cystoseira*, compensatory in *Phyllophora*, and largely buffering or beneficial in *Flabellia*.

Patterns in P: R ratios suggested a similar structure: while high light reduced metabolic efficiency overall, interaction terms again pointed toward taxon-specific and non-additive effects (formal *p*-values not reported due to mild violations of model assumptions). *Flabellia* and *Phyllophora* maintained autotrophic performance across conditions, while *Cystoseira* approached metabolic imbalance (P:R > = 1) under dual stress (Fig. 3). Respiration values contextualized this divergence: in *Cystoseira*, warming to medium temperature increased respiration substantially (–11.8 ± 0.52 nmol O_2_ m^−2^ s^−1^, Supplementary Fig. S-1), whereas gross photosynthesis failed to compensate, resulting in a steep decline in P:R. In contrast, *Phyllophora* experienced elevated respiration under medium temperature (e.g., − 13.5 ± 1.92 nmol O_2_ m^−2^ s^−1^, Supplementary Fig. S-1) but compensated via increased photosynthesis, maintaining high P:R. *Flabellia* again showed the greatest resilience, sustaining efficient P: R ratios across treatments despite variation in light and temperature.

Pairwise comparisons between taxa further supported these patterns. *Cystoseira* exhibited high sensitivity to stressor combinations. Under baseline conditions (control temperature, control light), net photosynthesis was 19.97 ± 1.67 nmol O_2_ m^−2^ s^−1^ (Fig. 2, Supplementary Table S-1), statistically indistinguishable from *Phyllophora* (19.72 ± 1.67 nmol O_2_ m^−2^ s^−1^, *p* = 0.994; Fig. 2, Supplementary Table S-2) but significantly higher than *Flabellia* (10.84 ± 1.83 nmol O_2_ m^−2^ s^−1^, *p* = 0.001; Fig. 2, Supplementary Table S-2). P: R was 3.64 ± 0.34, with respiration around − 7.6 ± 0.62 nmol O_2_ m^−2^ s^−1^ (Fig. 3; Supplementary Fig. S-1). Warming alone (medium temperature, control light) led to a decline in photosynthesis (12.43 ± 1.67 nmol O_2_ m^−2^ s^−1^; Fig. 2) and P: R (2.05 ± 0.19), alongside a marked increase in respiration (–11.8 ± 0.52 nmol O_2_ m^−2^ s^−1^). However, no pairwise contrasts were significant under this condition (Supplementary Table S-2). High light alone at control temperature (21 °C, 760 µmol photons m^−2^ s^−1^) caused a significant drop in photosynthesis to 7.75 ± 1.67 nmol O_2_ m^−2^ s^−1^ (Fig. 2), with pairwise comparisons showing that *Cystoseira* performed significantly worse than *Phyllophora* (*p* = 0.028; Supplementary Table S-2). P: R declined to 2.46 ± 0.23, with respiration at − 5.49 ± 0.49 nmol O_2_ m^−2^ s^−1^ (Supplementary Fig. S-1). Under dual stress (high temperature, high light), *Cystoseira’s* net photosynthesis dropped further to 2.59 ± 1.67 nmol O_2_ m^−2^ s^−1^ (Fig. 2), performing significantly worse than both *Flabellia* (*p* < 0.001, Supplementary Table S-2) and *Phyllophora* (*p* < 0.001; Supplementary Table S-2), with P: R reaching 1.32 ± 0.13. Respiration remained elevated (–10.3 ± 0.85 nmol O_2_ m^−2^ s^−1^), indicating a failure of photophysiological compensation under combined heat and light stress.

*Phyllophora* displayed moderate thermal tolerance and signs of compensation under dual stress. At baseline (control temperature, control light), *Phyllophora* did not differ from *Cystoseira* and exceeded *Flabellia* (*p* = 0.001), as previously described. P: R was 3.32 ± 0.31, with respiration at − 8.86 ± 0.67 nmol O_2_ m^−2^ s^−1^. *Phyllophora* maintained photosynthetic stability under medium warming and medium light (14.57 ± 1.67 nmol O_2_ m^−2^ s^−1^; Fig. 2, Supplementary Table S-2) and performed well under high light and high temperature (30 °C, 760 µmol photons m^−2^ s^−1^), as previously described. Under this condition, *Phyllophora* significantly outperformed *Cystoseira* (*p* < 0.001, Supplementary Table S-2), though it did not differ from *Flabellia* (*p* = 0.292; Fig. 2, Supplementary Table S-2). P: R remained high (3.66 ± 0.35, Fig. 3), with respiration moderate (–8.28 ± 0.63 nmol O_2_ m^−2^ s^−1^, Supplementary Fig. S-1), indicating that *Phyllophora* sustained net autotrophy via enhanced photosynthetic capacity, not lowered respiratory demand.

Observed trends in P: R ratios suggest that *Flabellia* was the most resilient taxon across all treatments. At baseline, *Flabellia* showed lower photosynthesis than the other taxa, as previously described. Respiration was also the lowest (–1.89 ± 0.24 nmol O_2_ m^−2^ s^−1^, Supplementary Fig. S-1), resulting in a P: R of 7.08 ± 0.73 (Fig. 3), which was the highest value observed under control conditions (Fig. 3). *Flabellia* reached peak performance under medium warming and medium light (26 °C, 320 µmol photons m^−2^ s^−1^), with photosynthesis rising to 17.59 ± 1.67 nmol O_2_ m^−2^ s^−1^ (Fig. 2) and P: R to 5.88 ± 0.56 (Fig. 3). While differences between *Phyllophora* and *Cystoseira* were not statistically significant in this condition (both *p* > 0.05, Supplementary Table S-2), *Flabellia* maintained highest efficiency across treatments based on observed P: R patterns. Under dual stress (high temperature, high light), *Flabellia* sustained high net photosynthesis (17.89 ± 1.67 nmol O_2_ m^−2^ s^−1^; Fig. 2), significantly outperforming *Cystoseira* (*p* < 0.001, Supplementary Table S-2), though not *Phyllophora* (*p* = 0.292; Supplementary Table S-2). Respiration remained moderate (–6.44 ± 0.50 nmol O_2_ m^−2^ s^−1^, Supplementary Fig. S-1), and P: R stayed high (3.75 ± 0.36, Fig. 3), supporting a stronger metabolic resilience.

## Discussion

We demonstrate clear taxon-specific variation in the physiological responses of Mediterranean macroalgae assemblages to temperature and light stress. While all three taxa experienced shifts in metabolic performance under altered conditions, the magnitude and direction of these changes differed substantially among genera. Combined increased temperature and light levels resulted in the strongest impairments, particularly in *Cystoseira*, where photosynthesis declined and respiratory demand increased, leading to metabolic imbalance. Our findings suggest that predicted climate-driven changes in temperature and light availability may affect individual taxa as well as alter competitive hierarchies and community structure within macroalgae assemblages.

Under baseline conditions, the brown alga *Cystoseira* had a similarly high net photosynthesis and respiration rate compared to the red alga *Phyllophora*, whereas the green alga *Flabellia* showed significantly lower photosynthesis and respiration rates. *Flabellia* is often categorized as an opportunistic or stress-tolerant species adapted to habitats with fluctuating environmental conditions. It invests less in high-rate metabolism and instead allocates more resources to resilience and persistence under variable conditions^33^. By contrast, *Cystoseira* are foundation species in stable, oligotrophic environments and have evolved high-efficiency carbon and nutrient assimilation systems to compete effectively for light and space^34,35^. *Phyllophora* are typically associated with similarly stable, clear-water habitats, though their metabolic traits are less well documented^36^. Elevated temperatures in interaction with light led to taxon-specific changes in photosynthetic and respiratory fluxes, supporting our hypothesis that stressor effects would interact, with the magnitude of impairment varying among taxa due to differences in thermal optima and metabolic flexibility. *Cystoseira* exhibited the steepest decline in net photosynthesis under medium warming (26 °C), accompanied by a marked increase in respiration, which reduced the P: R ratio to near metabolic imbalance. This impairment was most severe when warming coincided with high irradiance, suggesting that light was the dominant stressor while temperature exacerbated decline under dual stress. This pattern aligns with field reports of *Cystoseira* canopy degradation during marine heatwaves^3,37^, suggesting limited thermal compensation capacity coupled with low light tolerance. Photosynthetic and respiratory processes in algae rely on temperature-sensitive enzymes, particularly within Photosystem II. When temperatures exceed taxon-specific optima, these enzymes undergo denaturation, leading to a decrease in gross photosynthesis. For *Cystoseira*, optimal photosynthetic performance occurs between 15 and 25 °C, with physiological decline and early signs of enzyme destabilization reported above ~ 25 °C^35,38^. Compared to green and red algae, many brown algae also possess less effective protective mechanisms against combined light and thermal stress, including lower capacities for non-photochemical quenching (NPQ) and limited heat shock protein expression^39^, which likely constrain their ability to cope co-occuring high light and warming. In contrast, *Phyllophora* and *Flabellia* maintained higher photosynthetic stability with warming in interaction with light. *Phyllophora* showed moderate declines in P: R but compensated for elevated respiratory demand through increased gross photosynthesis, especially at high light stress, while *Flabellia* sustained high photosynthetic rates at medium and high light and low respiration across temperatures under these light levels. This interpretation is based on observed trends in P: R ratios and is further supported by statistically significant differences in net photosynthesis compared to the other taxa. These results suggest that red and green algae may sustain photosynthetic activity more effectively than brown canopy-formers under short-term thermal stress, potentially indicating a higher tolerance to elevated temperatures^12^. Many green algae possess active mechanisms for concentrating CO_2_ within their chloroplasts, known as carbon-concentrating mechanisms (CCMs), which help stabilize photosynthetic performance under suboptimal conditions such as heat stress^40,41^. At elevated temperatures, CO_2_ solubility in water decreases and Rubisco’s affinity for CO_2_ diminishes, but CCMs in green algae enable them to maintain sufficient inorganic carbon around Rubisco, sustaining photosynthesis even under thermal stress; this likely explains their relatively greater heat tolerance compared to brown algae, which typically possess less developed CCMs^42,43^. The combination of efficient CO_2_ acquisition and the ability to balance photosynthetic carbon gain with respiratory demand may explain why species such as *Phyllophora* and *Flabellia* maintained stable photosynthetic performance under elevated temperatures in our experiment, whereas *Cystoseira* exhibited pronounced sensitivity.

Light availability modulated photosynthetic performance in a taxon- and temperature-dependent manner, supporting our second hypothesis that photosynthetic performance would increase with light availability up to a taxon-specific threshold, beyond which photoinhibition would impair carbon assimilation. Evidence of photoinhibition at high irradiance was present in all taxa under some temperature conditions, but its magnitude and in some cases even its directionality varied with temperature. These differences may reflect variation in pigment composition and photoprotective mechanisms, suggesting differing light optima and tolerance thresholds across taxa. *Phyllophora* showed light responses that depended on temperature. Under control temperature, net photosynthesis declined at high irradiance, consistent with shade-adapted light harvesting; under warming, photosynthesis increased with high irradiance, indicating compensation at elevated light levels. As a red alga, *Phyllophora* possesses phycobiliproteins (e.g., phycoerythrin and phycocyanin) organized in phycobilisomes, which, along with the chlorophyll a common to all photosynthetic algae, facilitate efficient light harvesting in low-light environments by absorbing green to orange-red wavelengths. Within these complexes, the pigments efficiently transfer absorbed light energy to chlorophyll a, thereby optimizing photosynthesis under dim conditions^44^. Although it was shown that *Phyllophora* undergoes photoinhibition under high irradiance, as indicated by reduced efficiency of photosystem II (i.e., F_v_/F_m_) and oxygen evolution due to damage to its photosystem from blue light^45^, it may mitigate damage through the accumulation of UV-absorbing mycosporine-like amino acids (MAAs), which act as natural sunscreens and reduce photoinhibitory effects^46^. In contrast to the other taxa, *Flabellia* reached its peak photosynthesis at medium light when temperatures were elevated, whereas at control temperature rates remained low across light levels with only modest differences. As a green alga, *Flabellia* contains chlorophyll a, chlorophyll b, and carotenoids. Chlorophyll b broadens the absorption spectrum^47^, while carotenoids contribute to photoprotection by participating in the xanthophyll cycle, where they dissipate excess light energy and neutralize reactive oxygen species (ROS), thereby maintaining the functionality of Photosystem II under intense sunlight^48^. The observed peak in photosynthesis under medium light at elevated temperatures likely reflects saturation of the photosynthetic machinery without triggering photoinhibition, which is consistent with light-response curves reported in other subtropical green algae^49^. Among all tested algal taxa, *Cystoseira* exhibited the steepest reduction in net photosynthesis under high irradiance. In the brown alga *Cystoseira*, the pigment profile dominated by chlorophyll a, chlorophyll c, and fucoxanthin supports high photosynthetic efficiency under blue green light typical of mid depth environments^50^, but may be suboptimal under full-spectrum surface irradiance. This reduction is attributed to photoinhibition, evident in lowered F_v_/F_m_ values despite the presence of protective mechanisms such as non-photochemical quenching (NPQ) and antioxidant systems^51^. The absence of chlorophyll b and limited absorption in the red spectral range may further constrain its flexibility in light utilization.

The significant three-way interaction highlights that combined increases in temperature and irradiance produced taxon-specific responses, being non-additive and strongly negative in *Cystoseira* under dual stress, additive or synergistic in *Phyllophora*, and largely stabilizing in *Flabellia*. *Cystoseira* exhibited its highest metabolic efficiency, as reflected by the observed P: R ratio, under medium light and control or medium temperature, suggesting that under moderate irradiance and reduced oxidative pressure, this taxon can most effectively utilize its limited photoprotective capacity and light-harvesting potential. Under combined high temperature and high light, however, its P: R ratio declined to near unity, reflecting an imbalance between elevated respiratory demand and insufficient photochemical compensation. This collapse aligns with the restricted tolerance to combined warming and high irradiance and the reduced photoprotective capacity previously described for canopy-forming brown algae^39,51^. In contrast, *Phyllophora* maintained higher net photosynthesis and P: R ratios under combined high temperature and high light than under high temperature and medium light. Although red algae are typically considered shade-adapted, many species accumulate photoprotective compounds such as MAAs^46^; and exhibit efficient PSII repair mechanisms^45^. Together with moderate thermal tolerance^12^, these traits may explain the ability of *Phyllophora* to sustain, or even enhance, photosynthetic performance under dual stress. *Flabellia* consistently sustained high P: R ratios under both individual and combined stress conditions, confirming its robust performance. While a decline was observed under control temperature and high light compared to control temperature and control light, this was offset when light stress was coupled with medium temperature. This pattern suggests that moderate warming supports the activity of temperature-stable enzyme systems and CO_2_-concentrating mechanisms^40,42^, thereby stabilizing photosynthetic efficiency despite increased photoprotective costs. Carotenoid-based photoprotection further contributes to this resilience^48^. Taken together, these contrasting trajectories illustrate that co-occurring stressors accentuate species-specific physiological limits and adaptive strategies. While canopy-forming brown algae such as *Cystoseira* are most vulnerable when high irradiance coincides with warming, the persistence of red and green taxa underscores the potential for functional compensation within Mediterranean macroalgal communities.

The divergence in light and temperature stress tolerance among tested macroalgae taxa is likely to contribute to ongoing shifts in macroalgae communities^52,53^, which may become even more pronounced under future climate scenarios. Species with narrow physiological optima and low plasticity, such as *Cystoseira*, may face increasing exclusion from shallow, high-irradiance habitats during heatwaves. More tolerant species, such as *Flabellia* and *Phyllophora*, which, based on observed trends, maintain high photosynthetic efficiency under combined stress, may gain a competitive advantage. These dynamics may contribute to observed transitions from structurally complex, canopy-forming assemblages to simpler, fast-growing algal assemblages^54^. In particular, the spread of *Flabellia* may lead to a loss of structural complexity, reducing habitat availability and niche diversity for associated fauna. Simplified benthic structures may also impair key ecosystem functions such as sediment stabilization and energy transfer. Although *Flabellia* contributes to primary production and supports some epibionts^45,46^, it lacks the ecological engineering capacity of canopy-forming taxa, potentially resulting in more homogeneous and less resilient ecosystems. A reduction in structural heterogeneity may diminish habitat quality for associated species and alter benthic productivity, particularly if low P: R ratios under future climate scenarios limit carbon fixation and energy transfer to higher trophic levels. Projected warming in the Mediterranean by 2100, coupled with intensified stratification and nutrient limitation^5^, may further shift environmental baselines beyond the tolerance thresholds of sensitive taxa. Feedback loops such as sediment accumulation and light attenuation which could potentially be reinforced by dense algal mats could exacerbate recruitment failure in species like *Cystoseira*, increasing the risk of community shifts toward degraded or less diverse assemblages^55^.

Our analysis was conducted at genus level, which means that species-specific differences within *Cystoseira* were not considered. This limits the transferability of our results to individual species. Nevertheless, the genus-level approach is methodologically appropriate, since *Cystoseira* fulfills a consistent functional role as a structuring brown alga in Mediterranean habitats and is clearly distinct from the red and green algae examined here. Statistical tests using a mixed-effects model that included a random effect for taxon revealed negligible variance explained by this term and no improvement over a fixed-effects model, indicating limited variability within the sample pool. Descriptive checks further supported this conclusion, showing that *Cystoseira* displayed levels of dispersion comparable to or lower than the other taxa under most conditions, with greater variability only under the most stressful treatments. Taken together, these findings suggest that our results robustly capture functional differences among the three dominant taxa, even though species-specific patterns within *Cystoseira* should be addressed in more detail in future studies. The absence of empirical pigment dynamics, which are known to underpin stress responses in macroalgae, adds a limitation to true mechanistic insights in our study, even tough flux-based inference is a common proxy for photosynthetic responses^56,57^. While our experiment was designed to isolate temperature and light effects under controlled conditions, in situ responses are likely influenced by additional variables such as herbivory, seasonal irradiance cycles, and hydrodynamics. Future research should extend these findings using long-term, field-based experiments that integrate nutrient availability, acidification, and biotic interactions to evaluate community resilience and identify thresholds for functional collapse. Our findings provide experimentally grounded evidence that temperature and light interact to shape macroalgal metabolic performance in a taxon-specific manner, driving divergent stress responses that may signal future shifts in Mediterranean benthic communities. This highlights the urgent need for targeted monitoring and protection of light- and heat-sensitive primary producers such as *Cystoseira*, whose loss could trigger cascading ecological effects. As climate change accelerates, a mechanistic understanding of species-specific resilience will be essential for safeguarding the structure and function of coastal ecosystems.

## Supplementary Information

Below is the link to the electronic supplementary material.

Supplementary Material 1

## Data Availability

The datasets generated during and/or analysed during the current study are available in the corresponding GitHub Repository: https://github.com/MerlinWe/algae_photosynthesis.
