# Supplementary material for: Different photosynthetic responses to heat and light favour green and red over brown macroalgae in the mediterranean sea
Source: Sci Rep. 2025 Nov 26;15:42556. doi: 10.1038/s41598-025-28235-8 (PMC12663469; doi:10.1038/s41598-025-28235-8)
Supplement: Supplementary file 1 — Supplementary Material 1 [file 41598_2025_28235_MOESM1_ESM.pdf]

Different Photosynthetic Responses to Heat and Light Favour Green and Red over Brown Macroalgae in the Mediterranean Sea

Leonie Hesse[*1], Merlin Weiss[2], Selma D. Mezger[1], Yusuf C. El-Khaled[1,3], Benjamin Mueller[1], Alexandra Kler Lago[1], Mischa Schwarzmeier[4], Christian Wild[1]

Supplementary Material

Table S-1: *Parameter estimates from the best fitting model testing the effects of temperature, light, taxon, and interactions on net photosynthesis. Estimate and SE units are presented in* nmol O_2 m^{-2} s^{-1}. *Interaction terms represent the combined effects of multiple predictors, indicating deviations from additive effects.*

Parameter	Estimate	SE	T Ratio	p-value
(Intercept)	19.966	1.666	11.983	0.000
Temperature medium	-7.535	2.356	-3.198	0.002
Temperature high	-1.446	2.356	-0.614	0.540
Light medium	-3.612	2.356	-1.533	0.128
Light high	-12.213	2.356	-5.183	0.000
Genus *Flabellia*	-9.128	2.471	-3.694	0.000
Genus *Phyllophora*	-0.245	2.356	-0.104	0.917
Temperature medium : Light medium	10.369	3.332	3.112	0.002
Temperature high : Light medium	4.910	3.332	1.474	0.143
Temperature medium: Light high	1.001	3.332	0.300	0.764
Temperature high : Light high	-3.721	3.332	-1.117	0.266
Temperature medium: Genus *Flabellia*	7.819	3.415	2.290	0.024
Temperature high : Genus *Flabellia*	3.529	3.415	1.033	0.303
Temperature medium : Genus *Phyllophora*	-0.885	3.332	-0.266	0.791

Temperature high : Genus *Phyllophora*	-0.507	3.332	-0.152	0.879
Light medium : Genus *Flabellia*	0.694	3.415	0.203	0.839
Light high : Genus *Flabellia*	10.103	3.415	2.959	0.004
Light medium : Genus *Phyllophora*	0.224	3.332	0.067	0.946
Light high : Genus *Phyllophora*	6.361	3.332	1.909	0.058
Temperature medium : Light medium : Genus *Flabellia*	-0.982	4.771	-0.206	0.837
Temperature high : Light medium : Genus *Flabellia*	4.814	4.771	1.009	0.315
Temperature medium : Light high : Genus *Flabellia*	6.949	4.771	1.456	0.148
Temperature high : Light high : Genus *Flabellia*	10.804	4.771	2.264	0.025
Temperature medium : Light medium : Genus *Phyllophora*	-3.717	4.713	-0.789	0.432
Temperature high : Light medium : Genus *Phyllophora*	-1.876	4.713	-0.398	0.691
Temperature medium : Light high : Genus *Phyllophora*	11.842	4.713	2.513	0.013
Temperature high : Light high : Genus *Phyllophora*	13.242	4.713	2.810	0.006

Table S-2: *Pairwise comparisons of taxa under varying temperature and light conditions, estimated using marginal means from the final model. Estimate and SE units are presented in* nmol O_2 m^{-2} s^{-1}. *Tukey-adjusted p-values are reported to account for multiple comparisons.*

Contrast	Temperature	Light	Estimate	SE	DF	T Ratio	p-value
Cystoseira - Flabellia	control	control	9.128	2.471	134	3.694	0.001
Cystoseira - Phyllophora	control	control	0.245	2.356	134	0.104	0.994
Flabellia - Phyllophora	control	control	-8.883	2.471	134	-3.595	0.001
Cystoseira - Flabellia	medium	control	1.309	2.356	134	0.555	0.844
Cystoseira - Phyllophora	medium	control	1.130	2.356	134	0.480	0.881
Flabellia - Phyllophora	medium	control	-0.179	2.356	134	-0.076	0.997
Cystoseira - Flabellia	high	control	5.599	2.356	134	2.376	0.049
Cystoseira - Phyllophora	high	control	0.751	2.356	134	0.319	0.946
Flabellia - Phyllophora	high	control	-4.848	2.356	134	-2.057	0.103

Cystoseira - Flabellia	control	medium	8.433	2.356	134	3.579	0.001
Cystoseira - Phyllophora	control	medium	0.020	2.356	134	0.009	1.000
Flabellia - Phyllophora	control	medium	-8.413	2.356	134	-3.570	0.001
Cystoseira - Flabellia	medium	medium	1.596	2.356	134	0.677	0.777
Cystoseira - Phyllophora	medium	medium	4.622	2.356	134	1.962	0.126
Flabellia - Phyllophora	medium	medium	3.026	2.356	134	1.284	0.406
Cystoseira - Flabellia	high	medium	0.090	2.356	134	0.038	0.999
Cystoseira - Phyllophora	high	medium	2.403	2.356	134	1.020	0.566
Flabellia - Phyllophora	high	medium	2.313	2.356	134	0.982	0.590
Cystoseira - Flabellia	control	high	-0.975	2.356	134	-0.414	0.910
Cystoseira - Phyllophora	control	high	-6.116	2.356	134	-2.596	0.028
Flabellia - Phyllophora	control	high	-5.141	2.356	134	-2.182	0.078
Cystoseira - Flabellia	medium	high	-15.743	2.356	134	-6.681	0.000
Cystoseira - Phyllophora	medium	high	-17.073	2.356	134	-7.246	0.000
Flabellia - Phyllophora	medium	high	-1.330	2.356	134	-0.565	0.839
Cystoseira - Flabellia	high	high	-15.308	2.356	134	-6.497	0.000
Cystoseira - Phyllophora	high	high	-18.851	2.356	134	-8.001	0.000
Flabellia - Phyllophora	high	high	-3.544	2.356	134	-1.504	0.292

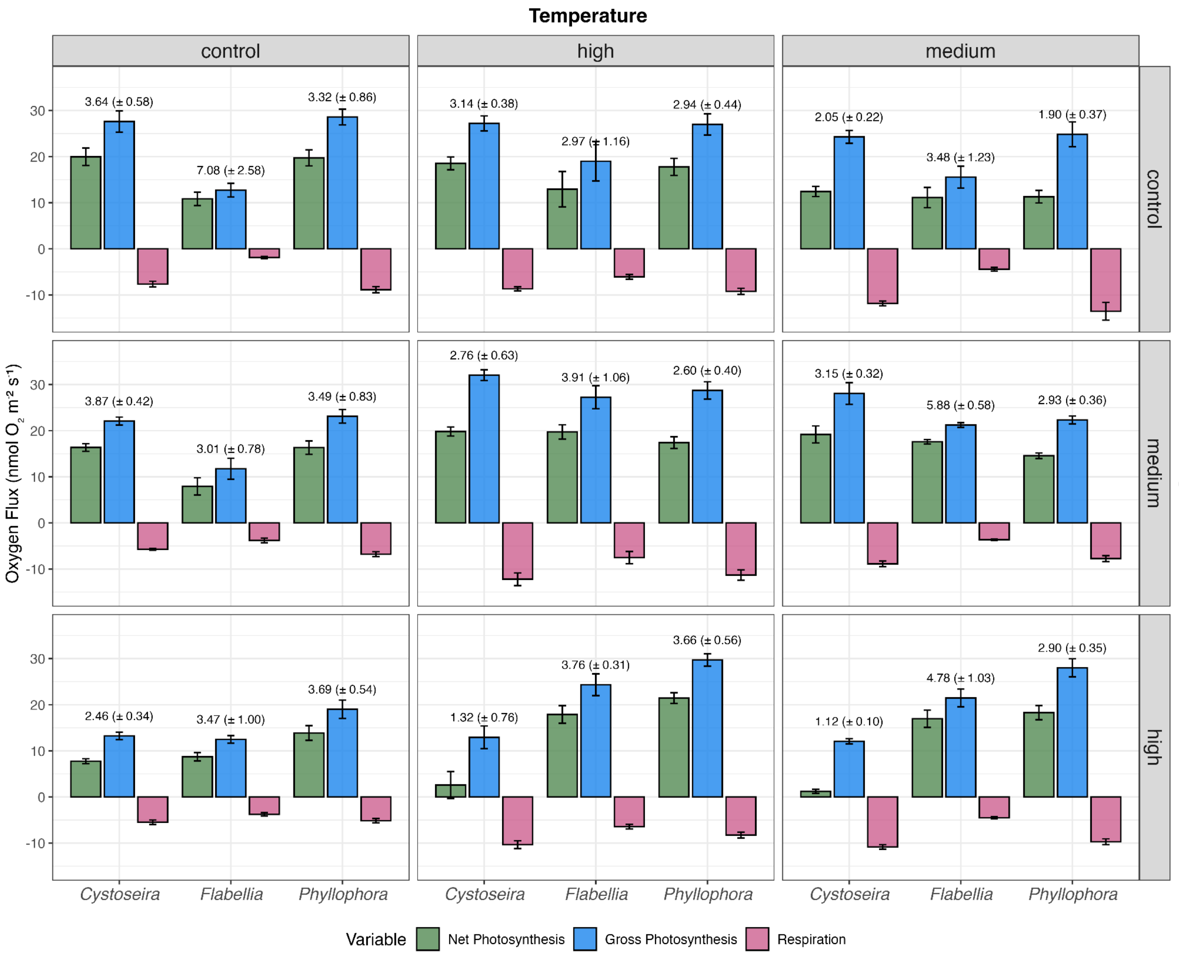

Figure S-1: Observed rates of net photosynthesis, gross photosynthesis, and respiration in Cystoseira, Phyllophora, and Flabellia under factorial combinations of temperature (control = 21 °C, medium = 26 °C, high = 30 °C) and light intensity (control = 180, medium = 320, high = 760 µmol quanta $m^{-2} s^{-1}$). Bars represent mean oxygen flux (nmol O_2 $m^{-2} s^{-1}$) ± standard deviation (SD). The blue bars indicate gross photosynthesis, green bars net photosynthesis, and pink bars respiration (negative values). Numeric values above the bars represent the corresponding photosynthesis-to-respiration (P:R) ratio calculated from raw means.
